# IRAK1 mediates TLR4-induced ABCA1 downregulation and lipid accumulation in VSMCs

**DOI:** 10.1038/cddis.2015.212

**Published:** 2015-10-29

**Authors:** L Guo, C-H Chen, L-L Zhang, X-J Cao, Q-L Ma, P Deng, G Zhu, C-Y Gao, B-H Li, Y Pi, Y Liu, Z-C Hu, L Zhang, Z-P Yu, Z Zhou, J-C Li

**Affiliations:** 1 Department of Neurology, Institute of Surgery Research, Daping Hospital, Third Military Medical University, Chongqing, People's Republic of China; 2 Department of Occupational Health, Faculty of Preventive Medicine, Third Military Medical University, Chongqing, People's Republic of China

## Abstract

The activation of Toll-like receptor 4 (TLR4) signaling has an important role in promoting lipid accumulation and pro-inflammatory effects in vascular smooth muscle cells (VSMCs), which facilitate atherosclerosis development and progression. Previous studies have demonstrated that excess lipid accumulation in VSMCs is due to an inhibition of the expression of ATP-binding cassette transporter A1 (ABCA1), an important molecular mediator of lipid efflux from VSMCs. However, the underlying molecular mechanisms of this process are unclear. The purpose of this study was to disclose the underlying molecular mechanisms of TLR4 signaling in regulating ABCA1 expression. Primary cultured VSMCs were stimulated with 50 *μ*g/ml oxidized low-density lipoprotein (oxLDL). We determined that enhancing TLR4 signaling using oxLDL significantly downregulated ABCA1 expression and induced lipid accumulation in VSMCs. However, TLR4 knockout significantly rescued oxLDL-induced ABCA1 downregulation and lipid accumulation. In addition, IL-1R-associated kinase 1 (IRAK1) was involved in the effects of TLR4 signaling on ABCA1 expression and lipid accumulation. Silencing IRAK1 expression using a specific siRNA reversed TLR4-induced ABCA1 downregulation and lipid accumulation *in vitro*. These results were further confirmed by our *in vivo* experiments. We determined that enhancing TLR4 signaling by administering a 12-week-long high-fat diet (HFD) to mice significantly increased IRAK1 expression, which downregulated ABCA1 expression and induced lipid accumulation. In addition, TLR4 knockout *in vivo* reversed the effects of the HFD on IRAK1 and ABCA1 expression, as well as on lipid accumulation. In conclusion, IRAK1 is involved in TLR4-mediated downregulation of ABCA1 expression and lipid accumulation in VSMCs.

Atherosclerosis is recognized as chronic inflammation of the arterial wall and is characterized by lipid-laden foam cell accumulation in inflammatory lesions.^1^ Oxidized low-density lipoprotein (oxLDL) has a major role in the development and progression of atherosclerosis and its complications. oxLDL has been reported to stimulate vascular smooth muscle cell (VSMC) proliferation and migration and to promote VSMC conversion to a macrophage-like state, leading to the expression of a variety of receptors for lipid uptake.^2, 3^ To date, co-staining with the macrophage marker CD68 and the VSMCs marker *α*-SMA has revealed that approximately 18% and 40% of CD68-positive cells originate as VSMCs in early and advanced human coronary atherosclerosis, respectively,^4^ indicating that many of the cells that are identified as macrophage-derived foam cells in human atherosclerosis are in fact derived from VSMCs. However, there is a striking lack of literature regarding the mechanism of VSMC-derived foam cell formation.

Using flow cytometric analysis and immunofluorescence staining methods, human VSMCs have been demonstrated to express Toll-like receptor 4 (TLR4) and the TLR4-related molecules, MD2 and CD14, but not TLR2.^5^ Upon stimulation, TLR4 activates the nuclear factor-*κ*B (NF-*κ*B) pathway and triggers the production of pro-inflammatory cytokines and chemokines, as well as the upregulation of cell surface molecules.^6^ Enhanced TLR4 signaling promotes atherosclerosis development and progression^7^ and induces VSMCs to switch from a contractile phenotype to a pro-inflammatory phenotype, resulting in the synthesis of MCP-1, IL-6, IL-*α* and so on.^8, 9, 10^ VSMCs are likely to engulf excess amounts of modified lipoproteins after phenotype conversion, transforming them into foam cells.^3, 11^ Comparatively, TLR4 knockout (TLR4^−/−^) significantly reduces cholesterol ester accumulation in mouse aortic SMCs.^12^ These results strongly indicate that TLR4 signaling influences lipid accumulation and induces pro-inflammatory effects in VSMCs. However, the available evidence is not sufficient to create definite conclusions, and future investigations are still needed to fully explore the detailed mechanisms of this process.

The balance between lipid accumulation from atherogenic lipoproteins and the removal of these lipids from cells is key to predicting the presence of foam cells in lesions. In this regard, scavenger receptors and ATP-binding cassette (ABC-) transporters have important roles in foam cell formation because they regulate lipid influx and efflux. Four members of the membrane ABC superfamily have been identified; ATP-binding cassette transporter A (ABCA) 1 and ABCG1 mediate cholesterol removal most efficiently from cells via the reverse cholesterol transport pathway.^13, 14^ In addition, the accumulation of serum lipids by VSMCs involves a macropinocytosis-like uptake pathway and is associated with ABCA1 downregulation.^15^ These studies suggest that ABCA1 downregulation may directly affect lipid accumulation in VSMCs, contributing to foam cell formation. The signaling pathways that are initiated by the pro-inflammatory cytokines IL-1*β* and tumor necrosis factor (TNF)-*α*, which are similar to TLR-dependent signaling,^16^ have been reported to upregulate low-density lipoprotein receptor-mediated cholesterol influx and to downregulate ABCA1-mediated cholesterol efflux *in vivo* and *in vitro.*^17^ Likewise, lipopolysaccharide (LPS) impairs ^3^H-cholesterol efflux from human macrophages to apolipoprotein A-I and significantly reduces macrophage expression of the cholesterol transporter ABCA1 *in vitro.*^18^ By pro-inflammatory and proatherogenic stimuli, NF-*κ*B is also involved in suppressing ABCA1 expression *in vivo* and *in vitro*, affecting lipid metabolism.^19, 20, 21^ However, the role and underlying mechanisms of TLR4 in regulating ABCA1 expression are unclear. Therefore, further studies are needed to clarify this issue and to better understand the role of TLR4 in lipid accumulation in VSMCs.

IL-1R-associated kinase 1 (IRAK1), an intracellular kinase, has been reported to positively regulate immune response. In addition, IRAK1 mediates the activation of NF-*κ*B and mitogen-activated protein kinase and has a critical role in the signaling cascades that are initiated by members of the TLR/interleukin-1 receptor (TLR/IL-1R) family and the tumor necrosis factor receptor superfamily.^22^ Elevated IRAK1 expression has been found in human atherosclerotic plaques.^23^ In monocytes, oxLDL induces IRAK1-dependent IL-1*β* transcription because significant inhibition of pro-IL-1*β* transcription is observed with IRAK1/4 inhibitor treatment.^24^ In addition, previous studies have indicated that IRAK1 is critically involved in the inhibition of cholesterol exporters expression and in the suppression of LPS-stimulated macrophage cholesterol efflux.^25, 26^ However, the effects of IRAK1 activity on TLR4 signaling-mediated cholesterol exporters have not been directly studied in any type of cell line. Therefore, the identification of this potential mechanism is of special importance for understanding the development of VSMC foam cells.

In the present study, we evaluated the potential role of TLR4 in VSMC lipid accumulation by manipulating TLR4 expression using wild-type (WT) and TLR4^−/−^ mice *in vivo* and genetic approaches *in vitro*. We also tested the hypothesis that IRAK1 was critical for TLR4-induced lipid transporter ABCA1 downregulation, which accelerated lipid accumulation in VSMCs.

## Results

### Enhanced TLR4 signaling downregulated ABCA1 expression *in vitro*

To test whether oxLDL stimulation enhanced TLR4 signaling, cultured VSMCs from thoracic aortas of WT and TLR4^−/−^ mice were stimulated with 50 *μ*g/ml oxLDL for 24, 48 or 72 h. A significant change in TLR4 expression was observed at 24 h. Compared with controls that were not treated with oxLDL, the average TLR4 mRNA level in the stimulated VSMCs was 2.3-fold higher (Supplementary Figure 2B), and the protein level was 3.2-fold higher (Supplementary Figure 2C). TLR4 is critical for activating the NF-*κ*B pathway and triggering the production of pro-inflammatory cytokines and chemokines.^6^ Using immunofluorescence, we observed a significant increase in NF-*κ*B p65 subunit translocation into nuclei after oxLDL treatment for 24 h (Supplementary Figure 3A). Accordingly, real-time PCR demonstrated that mRNA expression levels of IL-1*β* and TNF-*α* were significantly increased at 24 h post-oxLDL treatment in WT VSMCs (Supplementary Figures 3C and D). We also evaluated the mRNA level of MCP-1, which is known to be a strong responder to inflammatory stimuli and to be expressed in both VSMCs and macrophages. The results showed that MCP-1 mRNA expression in WT VSMCs increased by 3.3-fold after exposure to oxLDL for 24 h (Supplementary Figure 3E).

To further assess whether TLR4 was important for regulating downstream gene expression, we generated TLR4 deletion VSMCs from the thoracic aortas of C57BL/10ScNJ mice. Using immunofluorescence, we determined that NF-*κ*B p65 nuclear translocation was significantly inhibited in TLR4^−/−^ VSMCs that were treated with 50 *μ*g/ml oxLDL for 24 h (Supplementary Figure 3B). No changes in IL-1*β* and TNF-*α* mRNA expression levels were detected in TLR4^−/−^ VSMCs (Supplementary Figures 3C and D). Surprisingly, the MCP-1 mRNA expression in TLR4^−/−^ VSMCs was decreased by 70% (Supplementary Figure 3E).

**Figure 1:**
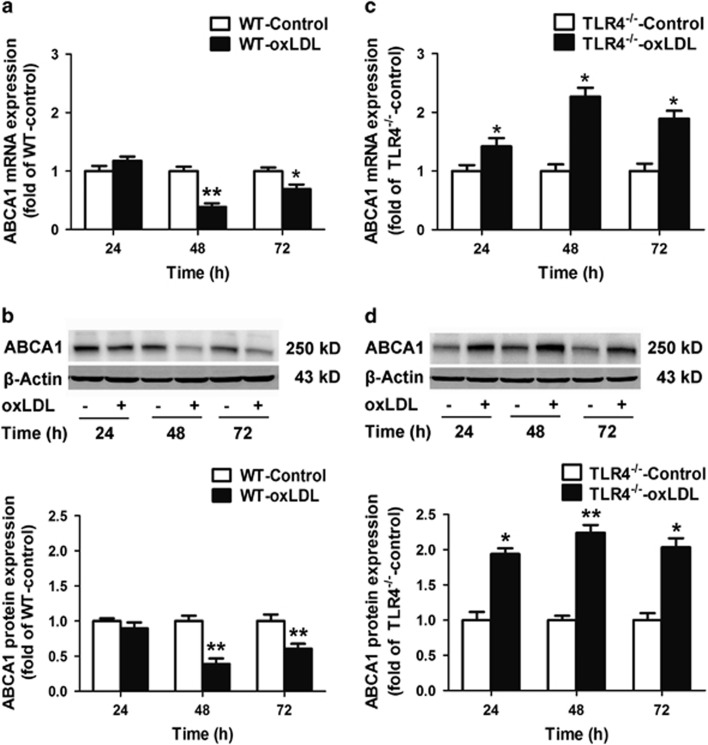
TLR4 knockout upregulated ABCA1 expression. Cultured WT or TLR4^−/−^ VSMCs were stimulated with or without 50 *μ*g/ml oxLDL for 24, 48 and 72 h. (**a** and **b**) Impaired expression of ABCA1 mRNA (*n*=4 experiments in duplicate) and protein (*n*=3 experiments in duplicate) was found in oxLDL-treated WT VSMCs. (**c** and **d**) Elevated expression of ABCA1 mRNA (**c**), *n*=4 experiments in duplicate), and protein (**d**), *n*=3 experiments in duplicate, were observed in TLR4^−/−^ VSMCs. For all experiments, the data are represented as the fold change relative to the control and are presented as the mean±S.E.M. *P<0.05, **P<0.01 compared with the untreated control group

To investigate whether enhanced TLR4 signaling affected the expression of the main cholesterol transporter ABCA1,^13^ cultured WT VSMCs were stimulated with 50 *μ*g/ml oxLDL for 24, 48 and 72 h. With continued oxLDL stimulation, ABCA1 expression was downregulated. At 48 h, both ABCA1 mRNA and protein expression decreased by 60% (Figures 1a and b).

### TLR4 deletion rescued ABCA1 downregulation and attenuated lipid accumulation in VSMCs *in vitro*

To further confirm that ABCA1 downregulation was TLR4 signaling dependent, cultured TLR4^−/−^ VSMCs were stimulated with 50 *μ*g/ml oxLDL for 24, 48 and 72 h. We determined that ABCA1 mRNA and protein expression were both significantly upregulated (Figures 1c and d). ABCA1 protein expression was 1.9–2.2 times that of controls that were not treated with oxLDL (Figure 1d).

We also used 100 ng/ml LPS and 50 *μ*g/ml native LDL to stimulate WT and TLR4^−/−^VSMCs for 48 h. We determined that in WT VSMCs, ABCA1 protein expression was decreased by 53 and 26% (Supplementary Figure 4A); however, in TLR4 deletion VSMCs, ABCA1 protein expression increased by 1.7- and 2.0-fold relative to control levels (Supplementary Figure 4B). These results indicated that, compared with LPS, oxLDL and native LDL had similar effects on ABCA1 expression. Furthermore, oxLDL had a more apparent effect on ABCA1 expression.

**Figure 2:**
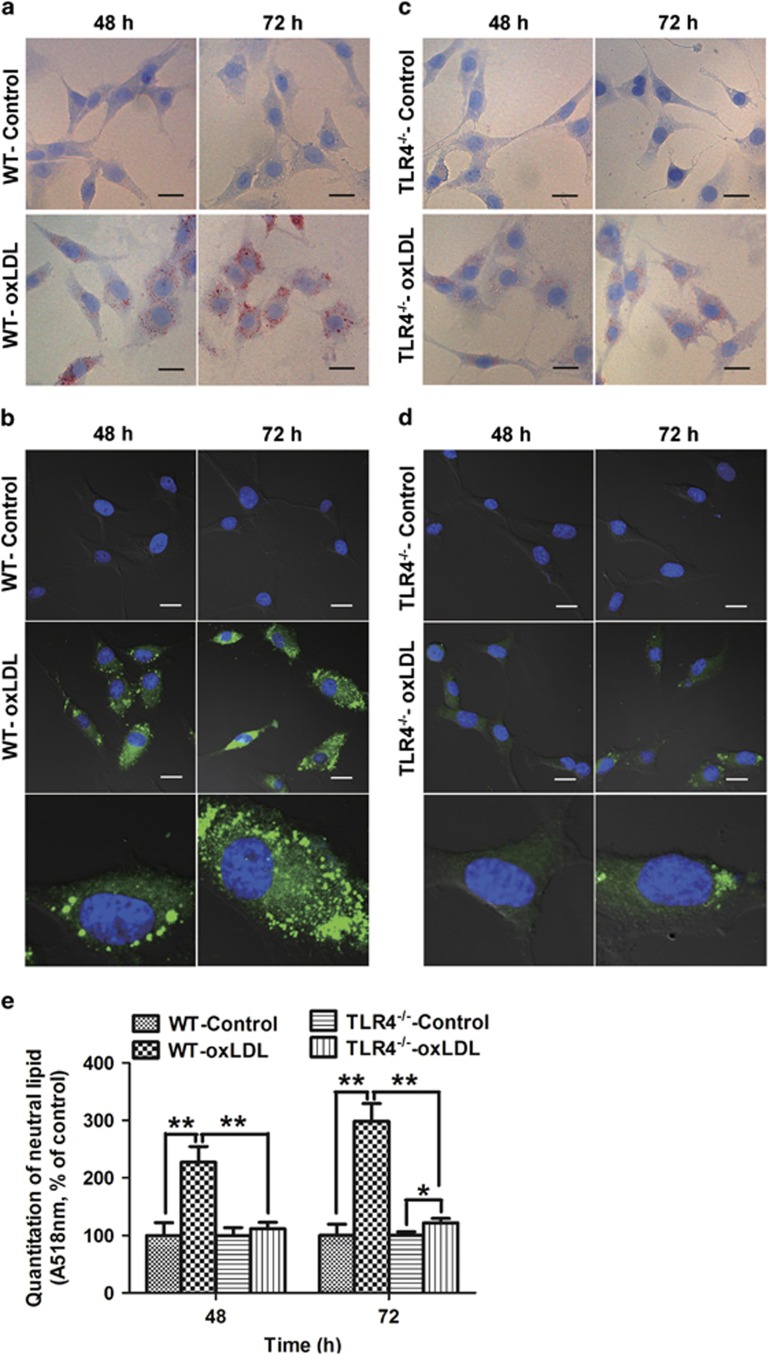
TLR4 knockout attenuated lipid accumulation in VSMCs *in vitro*. Cultured WT and TLR4^−/−^ VSMCs were treated with or without 50 *μ*g/ml oxLDL for 48 and 72 h. The cells were fixed with 4% paraformaldehyde and stained with Oil Red O or BODIPY 493/503 (green fluorescence). Cell nuclei were counterstained with hematoxylin (blue) or Hoechst 33342 (blue fluorescence). (**a** and **b**) Neutral lipid significantly accumulated in WT VSMCs. (**c** and **d**) Attenuated neutral lipid accumulation in TLR4^−/−^ VSMCs. (**e**) Quantification of lipid accumulation based on the OD values for destained Oil Red O (*n*=4 experiments in duplicate). Scale bar=20 *μ*m in all images. Data are represented as the fold change relative to the control and are presented as the mean±S.E.M. *P<0.05, **P<0.01 compared with the untreated control group

To investigate whether enhanced TLR4 signaling affected intracellular lipid loading, cultured WT and TLR4^−/−^ VSMCs were stimulated with 50 *μ*g/ml oxLDL for 48 and 72 h. Using Oil Red O staining, we determined that neutral lipids significantly accumulated in WT VSMCs and increased with time (Figure 2a). Similar results were observed using BODIPY 493/503 staining (Figure 2b). The absorbance values of the eluted Oil Red O solution in VSMCs were indicated via quantitative analysis of neutral lipid content relative to lipid accumulation in the cytoplasm. Therefore, we compared the absorbance values of eluted Oil Red O solution with a control at 518 nm. Based on the OD values for destained Oil Red O, the intracellular neutral lipid content of WT VSMCs increased to approximately 2.3 and 3.0 times the levels of controls treated without oxLDL at 48 and 72 h, respectively (Figure 2e). By comparison, lipid accumulation in TLR4^−/−^ VSMCs was significantly attenuated, as confirmed by Oil Red O staining, BODIPY 493/503 staining and by quantifying lipid accumulation based on OD values of destained Oil Red O (Figures 2c–e).

### IRAK1 is required for TLR4-regulated ABCA1 expression and lipid accumulation *in vitro*

**Figure 3:**
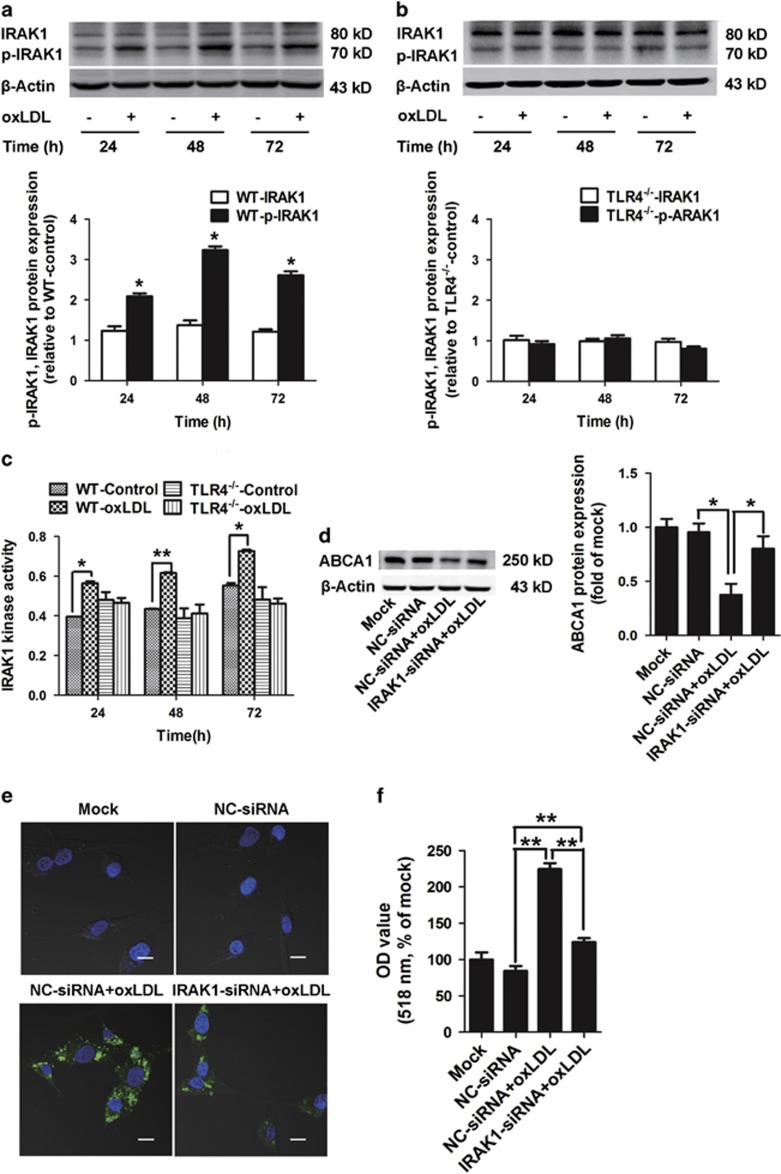
IRAK1 was involved in the effects of TLR4 signaling on VSMCs lipid accumulation and ABCA1 expression *in vitro*. WT and TLR4^−/−^ VSMCs were incubated with or without 50 *μ*g/ml oxLDL for 24, 48 and 72 h. (**a** and **b**) Western blot was used to show IRAK1 and p-IRAK1 protein expression in oxLDL-treated or untreated VSMCs. Increased expression of p-IRAK1 protein was detected in oxLDL-treated WT VSMCs (**a**, *n*=3 experiments in duplicate), but not in TLR4^−/−^ VSMCs (**b**, *n*=3 experiments in duplicate). (**c**) IRAK1 kinase activity was significantly elevated in WT VSMCs stimulated with oxLDL, but significantly inhibited in TLR4^−/−^ VSMCs in response to oxLDL, as determined by an *in vitro* kinase assay (*n*=2 experiments in duplicate). (**d**–**f**) WT VSMCs were transfected with IRAK1-specific siRNA using a transfection reagent. A non-related scrambled siRNA was used as a negative control (NC-siRNA). At 24 h following transfection, the cells were exposed to 50 *μ*g/ml oxLDL for 48 h. IRAK1 deficiency upregulated ABCA1 expression (**d**, *n*=3 experiments in duplicate) and attenuated lipid accumulation in WT VSMCs as determined by BODIPY 493/503 staining (**e**, green fluorescence, scale bar=20 *μ*m). Quantification of lipid accumulation was based on the OD values for destained Oil Red O in WT VSMCs (**f**, *n*=4 experiments in duplicate). All data are represented as the fold change relative to the controls and are expressed as the mean±S.E.M. *P<0.05, **P<0.01 compared with the untreated control group

To gain further insight into the signaling mechanism responsible for TLR4-mediated downregulation of ABCA1 expression and its promotion of lipid accumulation in VSMCs, we examined the involvement of IRAK1 activity. In WT VSMCs treated with 50 *μ*g/ml oxLDL, we measured IRAK1 activation by performing phospho-blotting. A significant increase in IRAK1 phosphorylation at Thr387 (p-IRAK1) was detected that increased by 2.1-fold at 24 h, and the maximum induction was 3.2-fold higher after treating with oxLDL for 48 h (Figure 3a). By comparison, IRAK1 activity was notably inhibited in TLR4^−/−^ VSMCs (Figure 3b). It is well known that IRAK1 activation is often accompanied by its degradation;^27^ therefore, we examined IRAK1 kinase activity in WT and TLR4^−/−^ VSMCs in the presence or absence of oxLDL for 24, 48 and 72 h. The results demonstrated that IRAK1 kinase activity was significantly elevated in oxLDL-pretreated WT VSMCs, but not in TLR4^−/−^ VSMCs (Figure 3c).

To ascertain the key role of IRAK1 in TLR4-induced ABCA1 downregulation and VSMC lipid accumulation, IRAK1-, MyD88-, IRAK2- and IRAK4-specific siRNAs were used for gene silencing. The knockdown of IRAK1, IRAK2, IRAK4 and MyD88 mRNA levels by siRNA were measured by real-time PCR (Supplementary Figure 5). We determined that knockdown of MyD88, IRAK2 and IRAK4 gene expression did not reverse the reduced expression of ABCA1 in WT VSMCs in response to oxLDL (Supplementary Figure 6). However, inhibition of IRAK1 gene expression significantly upregulated ABCA1 expression in oxLDL-treated WT VSMCs (Figure 3d) and, notably, attenuated lipid accumulation in WT VSMCs as determined by BODIPY 493/503 staining (Figure 3e). These results were further confirmed by the absorbance values that were obtained for destained Oil Red O (Figure 3f). Collectively, these *in vitro* results suggest that IRAK1 activity is involved in TLR4-mediated downregulation of ABCA1 expression and inducing lipid accumulation in VSMCs.

### Enhanced TLR4 signaling via the administration of a high-fat diet (HFD) activated IRAK1 and downregulated ABCA1 *in vivo*

**Figure 4:**
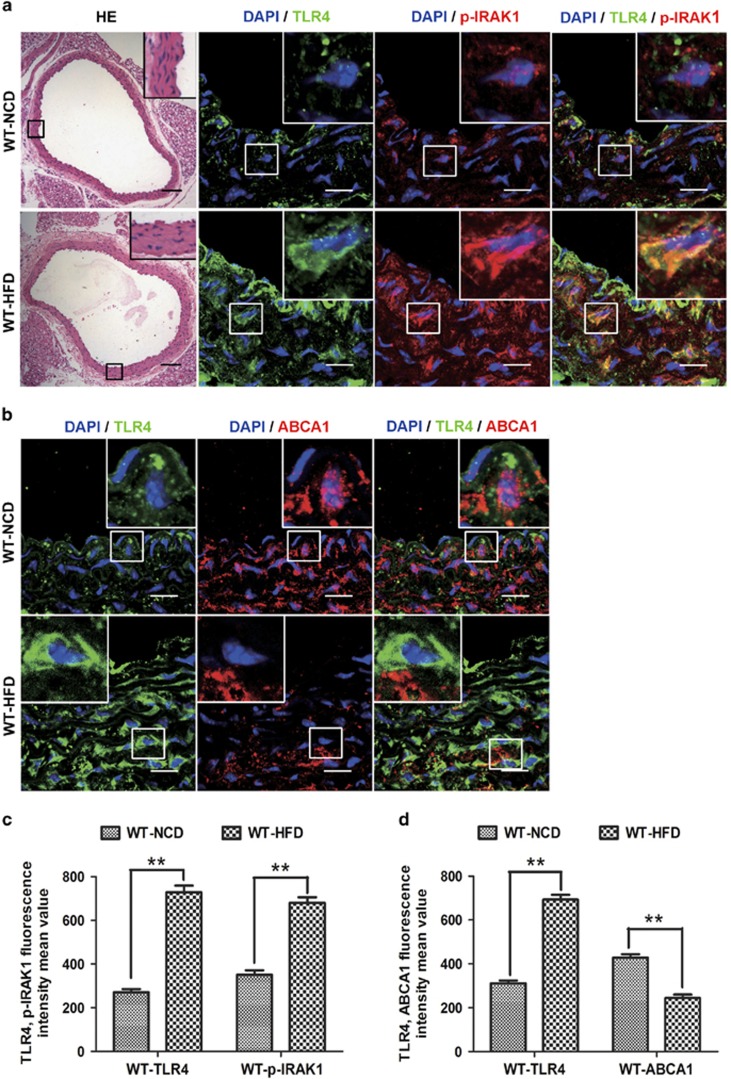
Effects of TLR4 signaling induced by a high-fat diet (HFD) on IRAK1 activity and ABCA1 expression *in vivo*. WT mice were either fed a normal chow diet (NCD) or a very high-fat diet for 12 weeks and then their thoracic aortas were harvested. (**a**) Hematoxylin and eosin (HE) staining on cross-sections from thoracic aorta were presented. Artery sections were double-stained with TLR4 (green fluorescence) and p-IRAK1 (red fluorescence) antibodies and co-localization was determined in the merged images. (**b**) Immunohistochemical studies were performed by co-staining TLR4 (green fluorescence) and ABCA1 (red fluorescence). The merged panel indicates the co-localization of TLR4 with ABCA1. In images **a** and **b**, the nuclei were stained with DAPI (blue fluorescence). Scale bar in HE images=100 *μ*m; scale bar in immunofluorescence images=20 *μ*m. (**c** and **d**) The p-IRAK1 or ABCA1 fluorescence intensity mean values were determined by the mean±S.E.M. of *n*=6 non-consecutive sections from five mice. **P<0.01

To confirm the above data from *in vitro* experiments, we evaluated the effects of initiating TLR4 signaling via the administration of a HFD on IRAK1 activity and ABCA1 expression *in vivo*. VSMCs are the major cell type in the medial layer of the vessel wall, and a significant number of VSMCs exist within the intima as well.^2^ Using immunofluorescence, we further demonstrated that both ABCA1 and p-IRAK1 co-localized with TLR4 in medial VSMCs. Furthermore, we observed that the administration of a HFD noticeably induced TLR4 and p-IRAK1 expression in medial VSMCs (Figures 4a and c); however, the level of ABCA1 expression was decreased by 43% (Figures 4b and d) compared with a normal chow diet (NCD) group.

### TLR4 knockout inhibited HFD-induced IRAK1 activation and rescued ABCA1 expression *in vivo*

**Figure 5:**
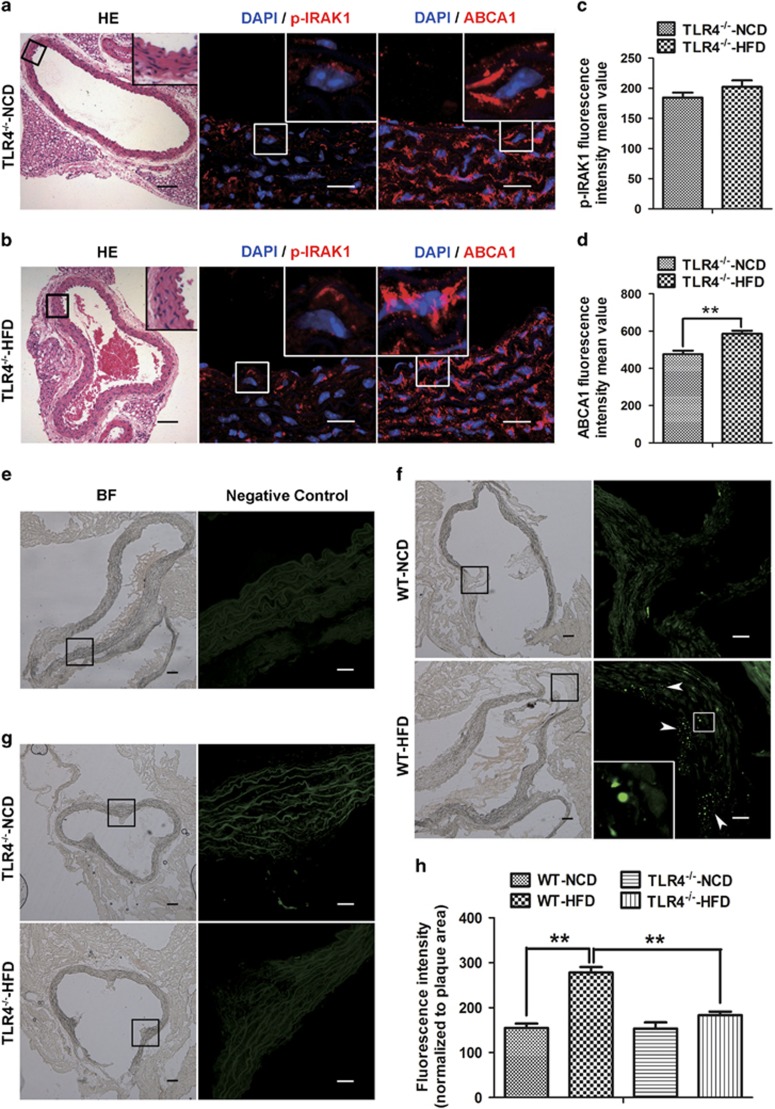
*In vivo* TLR4 knockout inhibited high-fat diet (HFD)-induced IRAK1 activation and rescued ABCA1 downregulation. (**a** and **b**) TLR4^−/−^ mice were fed either a normal chow diet (NCD) or a very HFD for 12 weeks. Following this, their thoracic aortas were obtained. Thoracic aortas were sectioned, and stained for hematoxylin and eosin. IRAK1 activation (red fluorescence) and ABCA1 expression (red fluorescence) were determined using immunofluorescence staining. DAPI was used for nuclear counterstaining (blue fluorescence). (**c** and **d**) Determination of p-IRAK1 and ABCA1 fluorescence intensity mean values (*n*=6 non-consecutive sections from 5 mice). (**e**–**g**) WT and TLR4^−/−^ mice were fed either a normalized chow diet or a very HFD for 12 weeks. Following this, aortic root sections were obtained. A negative control was performed using phosphate-buffered saline (PBS) instead of primary antibodies. BF (bright field) images show cross-sections of the arteries from which the immunofluorescence images were obtained (**e**). Detection of lipid accumulation in aortic roots using BODIPY 493/503 staining (green fluorescence). Significant lipid accumulation was observed in aortic root sections from WT mice fed a HFD (**f**), but not from TLR4^−/−^ mice fed a HFD (**g**). The white arrows show representative lipid accumulation. (**h**) Determination of fluorescence (normalized to plaque area, *n*=6 non-consecutive sections from 5 mice). Scale bar in BF and HE images=100 *μ*m; scale bar in immunofluorescence images=20 *μ*m. Data are expressed as the mean±S.E.M. **P<0.01 compared with NCD group

To further ascertain whether TLR4 signaling regulated ABCA1 downregulation and lipid accumulation via IRAK1 activity *in vivo*, we studied TLR4^−/−^ mice that were fed with either a NCD or a very HFD. Compared with TLR4^−/−^ mice on a NCD, the TLR4^−/−^ mice that were fed a HFD had significantly inhibited IRAK1 activity (Figures 5a and c), and the level of ABCA1 expression was slightly increased by 20% as confirmed by mean fluorescence intensity (Figures 5b and d). Using BODIPY 493/503 staining, neutral lipids were determined to accumulate in the aortic roots of WT mice on HFDs (Figure 5f), but not in TLR4^−/−^ mice on HFDs (Figure 5g). Lipid content was further quantified by fluorescence intensity (Figure 5h).

Collectively, our *in vivo* results further suggest that enhanced TLR4 signaling because of HFD-induced IRAK1 activation and ABCA1 downregulation, leading to lipid accumulation in the aortic root. TLR4 knockout attenuated lipid accumulation in mouse aortic roots by inhibiting IRAK1 activation and upregulating ABCA1 expression.

## Discussion

In the present study, we revealed a promotion effect of TLR4 on lipid accumulation in VSMCs as well as the underlying molecular mechanisms of this process. First, we demonstrated that VSMC lipid accumulation and ABCA1 downregulation were markedly dependent on TLR4 signaling *in vivo* and *in vitro*. Second, TLR4 knockout significantly reversed ABCA1 downregulation and attenuated lipid accumulation in VSMCs *in vivo* and *in vitro*. Third, we determined that IRAK1 activity played a critical role in TLR4-regulated ABCA1 expression and lipid accumulation.

A well-known function of ABCA1 is the removal of excess lipids from cells via lipid-free apolipoprotein A-I on the cell surface. Emerging studies report that human atherosclerotic lesion SMCs exhibit impaired ABCA1 expression.^4, 14, 28^ With lipid loading in a proatherogenic milieu, VSMCs go on to downregulate ABCA1 expression.^14, 29^ In addition, previous studies have indicated that LPS can suppress macrophage ABCA1 expression and inhibit cholesterol export.^26, 30^ However, the effects of TLR4 signaling when initiated by oxLDL on ABCA1-mediated lipid accumulation in VSMCs are largely unknown. Our current study addressed this issue and was the first to directly demonstrate that enhancing TLR4 signaling using a HFD or oxLDL impaired ABCA1 expression, leading to excess lipid accumulation in VSMCs as confirmed by Oil Red O staining and BODIPY 493/503 staining. It is known that decreased ABCA1 levels may lead to the activation of TLR4 signaling and elevate the expression of inflammatory cytokines.^31, 32^ Surprisingly, TLR4 knockout led to significantly upregulated ABCA1 expression *in vivo* and *in vitro*. A previous study reported that peroxisome proliferator-activated receptor *γ* (PPAR*γ*) has a crucial role in the induction of ABCA1 expression and promotes cholesterol removal from macrophages via liver X receptor *α* (LXR*α*).^33^ In addition, upon activation of TLR4 by LPS, NF-*κ*B drives down macrophage PPARγ expression.^34^ Therefore, inhibition of the expression of LXR*α*/PPAR*γ* may be a potential mechanism that is involved in TLR4/IRAK1/NF-κB-mediated ABCA1 downregulation. TLR4 knockout may induce elevated expression of LXR*α*/PPAR*γ* and rescue the downregulation of ABCA1. Further studies are needed to fully explore the details of this mechanism.

TLR4 signaling is thought to be initiated by ligand binding or trafficking to lipid rafts.^35^ Furthermore, TLR4 signaling is critical for NF-κB nuclear translocation in gastric cancer.^36^ Upon stimulation, NF-κB translocates to nuclei and stimulates gene expression, including that of pro-inflammatory cytokines and co-stimulatory molecules.^37^ The activation of NF-*κ*B and induced production of pro-inflammatory cytokines can be used as a functional readout for the activation of TLR4.^38^ TLRs recognize conserved pathogen-associated molecular patterns and induce innate immune responses that are essential for host defenses. oxLDL is regarded as an endogenous ligand that can be recognized by pattern recognition receptors, such as scavenger receptor CD36 or TLRs.^39, 40^ These findings suggest that TLR4 signaling can be directly activated by oxLDL stimulation. In agreement with our findings, oxLDL pre-treatment significantly enhanced TLR4 signaling and induced NF-*κ*B p65 nuclear translocation in WT VSMCs. Increased expression of IL-1*β*, TNF-*α* and MCP-1 was also observed in oxLDL-treated WT VSMCs. By contrast, no changes to IL-1*β* mRNA and TNF-*α* mRNA expression were found; however, significantly decreased expression of MCP-1 mRNA was detected in TLR4 knockout VSMCs in response to oxLDL. It is likely that, with the exception of TLR4 signaling, some other pathways may be involved in mediating the expression of pro-inflammatory cytokines, such as IL-1*β*, but not MCP-1. Minimally modified LDL, a very early form of oxLDL, primes macrophages for NLRP3 inflammasome activation. In addition, the activation of NLRP3 inflammasome has been reported to induce caspase-1 cleavage and IL-1*β* release in cholesterol crystals that accumulate in macrophages.^41^ However, to fully clarify this issue, further studies are needed.

Previous studies have demonstrated that TLRs and IL-1R family members share a conserved stretch of ~200 amino acids in their cytoplasmic region, known as the ‘TLR- and IL-1R related' domain. IL-1R recognizes the cytokine IL-1, which is produced by several cell types in response to various stimuli. IRAK1 has been implicated in signal transduction of the TLR/IL-1R family.^22^ To further assess the signal transduction mechanisms of TLR4, we determined that IRAK1 was critical for TLR4 signaling-induced ABCA1 downregulation and lipid accumulation in VSMCs *in vitro*. This result was further confirmed by siRNA silencing of IRAK1 expression in WT VSMCs and by studying WT and TLR4^−/−^ mice that were fed either a NCD or a HFD *in vivo*. Notably, IRAK1 phosphorylation is needed for downstream signaling but rapidly results in autophosphorylation, which eventually leads to degradation.^27^ Therefore, we further examined IRAK1 kinase activity. A previous study has indicated that the kinase activity of IRAK1 is dispensable for activation of a 165-nucleotide element-mediated post-transcriptional mechanism and that IRAK1 only acts as an adapter.^42^ However, another study highlighted the importance of IRAK1 kinase activity to effects mediated by IL-1*β*.^43^ Our study evaluated the key role of IRAK1 kinase activity in TLR4 signaling-mediated ABCA1 downregulation and VSMC lipid accumulation. Furthermore, several cytokines and chemokines were expressed in lipid-laden WT VSMCs in our study. Pro-inflammatory molecules, such as IL-1, are reported to induce IRAK1 phosphorylation, which is needed for its own kinase activity.^44, 45^ This may be a reason for the continued elevated expression of active IRAK1 that was observed by phospho-blotting in our study.

In conclusion, our findings provide the first evidence that IRAK1 has an important role in TLR4 signaling, which downregulates ABCA1 expression and promotes lipid accumulation in VSMCs. TLR4 deletion reversed ABCA1 downregulation and attenuated lipid accumulation in VSMCs. We therefore provided further insight into VSMC-derived foam cell formation and offered IRAK1 as a promising target for the prevention and treatment of atherosclerosis.

## Materials and Methods

### Animals

Male TLR4^−/−^ mice (stock number: 003752) and WT mice (stock number: 000664) were obtained at 4–6 weeks of age from the Jackson Laboratories (Bar Harbor, ME, USA). Both WT and TLR4^−/−^ mice were randomly divided into two groups (*n*=12 mice per group), which either group received a NCD (protein 18.3%, fat 10.2%, carbohydrates 71.5%, D12450B, Research Diets, New Brunswick, NJ, USA) or a very HFD (protein 18.1%, fat 61.6%, carbohydrates 20.3%, D12492, Research Diets) for 12 weeks. Following this, the mice were anesthetized with an intraperitoneal injection of 60 mg/g body weight of sodium pentobarbital. After anesthesia, thoracic aortas and hearts were harvested. Animal care and procedures conformed to the Guide for the Care and Use of Laboratory Animals.

### Cell culture

VSMCs were isolated from thoracic aortas of WT mice and TLR4^−/−^ mice using an explant technique and were cultured in DMEM supplemented with 10% FBS, 100 U/ml penicillin and 100 mg/ml streptomycin in air supplemented with 5% CO_2_ at 37 °C. To verify that the cultured cells were VSMCs, and not myofibroblasts, immunocytochemical localization of smooth muscle-specific *α*-actin and myofibroblast marker were performed using anti-*α*-SMA, anti-SM-22*α* and anti-vimentin antibodies. The purity of the cells was found to be >95% (Supplementary Figure 1). Cells in the second to sixth passages were used for experiments. oxLDL (Yiyuan, Guangdong, China) was used to induce lipid accumulation in VSMCs. To date, thiobarbituric acid-reactive substances have been determined colorimetrically using malondialdehyde (MDA) as a standard. The starting LDL was 0.1–0.2 nmol of MDA/mg protein and oxLDL was 18–23 nmol of MDA/mg protein. For gene silencing, siRNAs targeting IRAK1, IRAK2, IRAK4 or MyD88 or a non-related scrambled siRNA (Santa Cruz Biotechnology, Santa Cruz, CA, USA) were transfected into WT VSMCs. Experiments were conducted 24 h after transfection.

### Real-time PCR

Total RNA was extracted using RNAiso Plus (Takara Bio, Otsu, Japan). cDNA was obtained by reverse-transcription PCR. Real-time PCR was performed on a CFX96 Real-Time System (Bio-Rad, Hercules, CA, USA) using SYBR Premix Ex TaqTMII (Takara Bio). The primers for TLR4, TLR3, TLR2, TLR6, CD36, ABCA1, TNF-*α*, IL-1*β*, MCP-1 and *β*-actin amplification are shown in Supplementary Table 1. The target gene expression level was normalized to *β*-actin and was presented as a fold change using the formula 2^−ΔΔCt^.

### Western and phospho-blot analysis

Cultured VSMCs were lysed in RIPA buffer (Thermo Scientific, Waltham, MA, USA), and protein concentration was determined. Protein samples were separated using SDS-PAGE and were transferred to a polyvinylidene difluoride membrane. Following this, the membranes were incubated with primary antibodies against TLR4, ABCA1 (Abcam, Burlingame, CA, USA), IRAK1 (Santa Cruz Biotechnology) or p-IRAK1 (phospho-Thr387, Biorbyt, Cambridge, UK). After incubation with an appropriate secondary antibody, proteins were detected using enhanced chemiluminescence (Pierce, San Diego, CA, USA) and quantified using a Chemi Doc XRS^+^ Imager (Bio-Rad).

### Immunofluorescence staining

Serial fresh-frozen sections (~10 *μ*m) and cultured VSMCs were stained with primary antibodies against TLR4 (1 : 50 dilution), p-IRAK1 (1 : 50 dilution) and ABCA1 (1 : 50 dilution). Following this, Alexa Fluor 555- and 647-labeled donkey anti-mouse and donkey anti-rabbit secondary antibodies (Life Technology, Carlsbad, CA, USA) were used for visualization. Cell nuclei were stained with Hoechst 33342 (Life Technology) or DAPI (Life Technology). The images were collected using a Zeiss confocal laser scanning microscope (Carl Zeiss, LSM 510 Meta Confocal Laser Scanning Microscope, Jena, Germany).

### Lipid accumulation assay

Neutral lipid accumulation in VSMCs and aortic roots was determined using Oil Red O (Sigma-Aldrich, St. Louis, MO, USA) staining or BODIPY 493/503 (Molecular Probes, Eugene, OR, USA) staining. Lipid content was quantified by isopropanol extraction of Oil Red O from stained cells and optical density determinations at 518 nm as previously described,^46, 47^ or by fluorescence intensity.

### *In vitro* IRAK1 kinase activity assay

IRAK1 kinase activity was measured as previously described^43, 48^ with some modifications. Briefly, cells from the different experimental groups were washed with kinase assay buffer (20 mM MOPS (pH 7.2), 50 mM MgCl_2_, 2 mM EGTA, 1 mM DTT) and then were lysed in 0.1% Nonidet P-40 lysis buffer (50 mM Tris-Cl (pH 8.0), 137 mM sodium chloride, 2 mM EDTA, 5% glycerol, 0.1% Nonidet P-40). The lysates were centrifuged at 16 000 r.p.m. for 5 min, and the supernatants were collected. Protein concentration was measured. The reaction buffer was comprised of 5 *μ*g MBP substrate, 0.5 mM ATP and 10 *μ*Ci [γ-^32^P] ATP, and was incubated with the samples for 30 min at 30 °C. Following this, each sample (10 *μ*l) was added to 190 *μ*l reaction buffer in a 96-well plate (*μ*Clear, Greiner, Germany). IRAK1 kinase activity was measured as a change in optical density at 340 nm (ΔOD340) at 30 °C for 5 min. The activity of IRAK1 kinase was expressed as ΔmOD_340_/min/mg protein (ΔmOD_340_=ΔOD_340_/1000).

### Statistics

All values reported are the mean±S.E.M. Two-group comparisons were performed using a *t*-test for independent samples. Multiple-group statistical analyses were performed using one-way ANOVA followed by Fisher's *post hoc* tests. Statistics were calculated using the GraphPad Prism 5 software package (La Jolla, CA, USA). *P*<0.05 was considered to be statistically significant.

## Abbreviations

TLR4: Toll-like receptor 4
IRAK1: IL-1R-associated kinase 1
ABCA1: ATP-binding cassette transporter A1
VSMCs: vascular smooth muscle cells
oxLDL: oxidized low-density lipoprotein
NF-*κ*B: nuclear factor-*κ*B
LPS: lipopolysaccharide
PPAR*γ*: proliferator-activated receptor *γ*
LXR*α*: liver X receptor *α*
MDA: malondialdehyde
NCD: normal chow diet
HFD: high-fat diet
