# Supplementary figures and images for: IRAK1 mediates TLR4-induced ABCA1 downregulation and lipid accumulation in VSMCs
Source: Cell Death Dis. 2015 Oct 29;6(10):e1949–. doi: 10.1038/cddis.2015.212 (PMC5399175; doi:10.1038/cddis.2015.212)

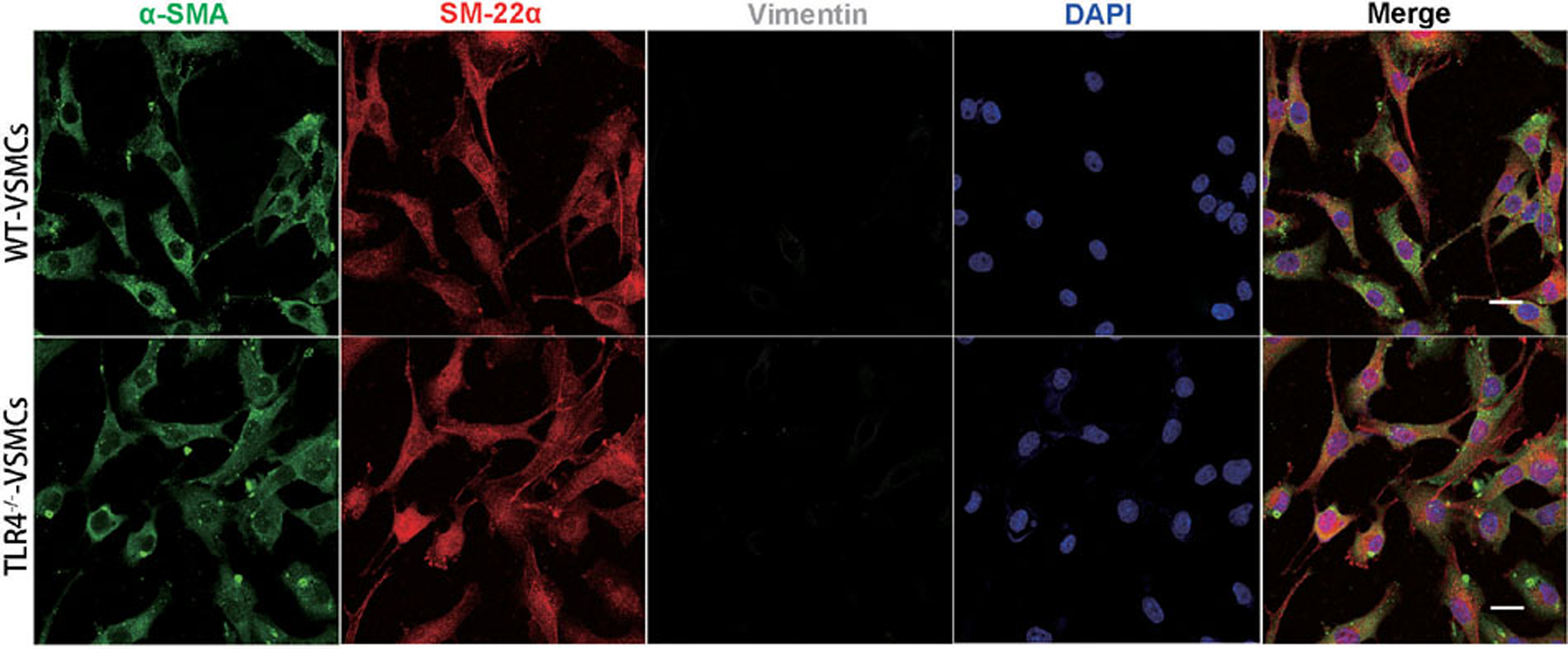

Supplement: Supplementary Figure 1 [file cddis2015212x1.tif]

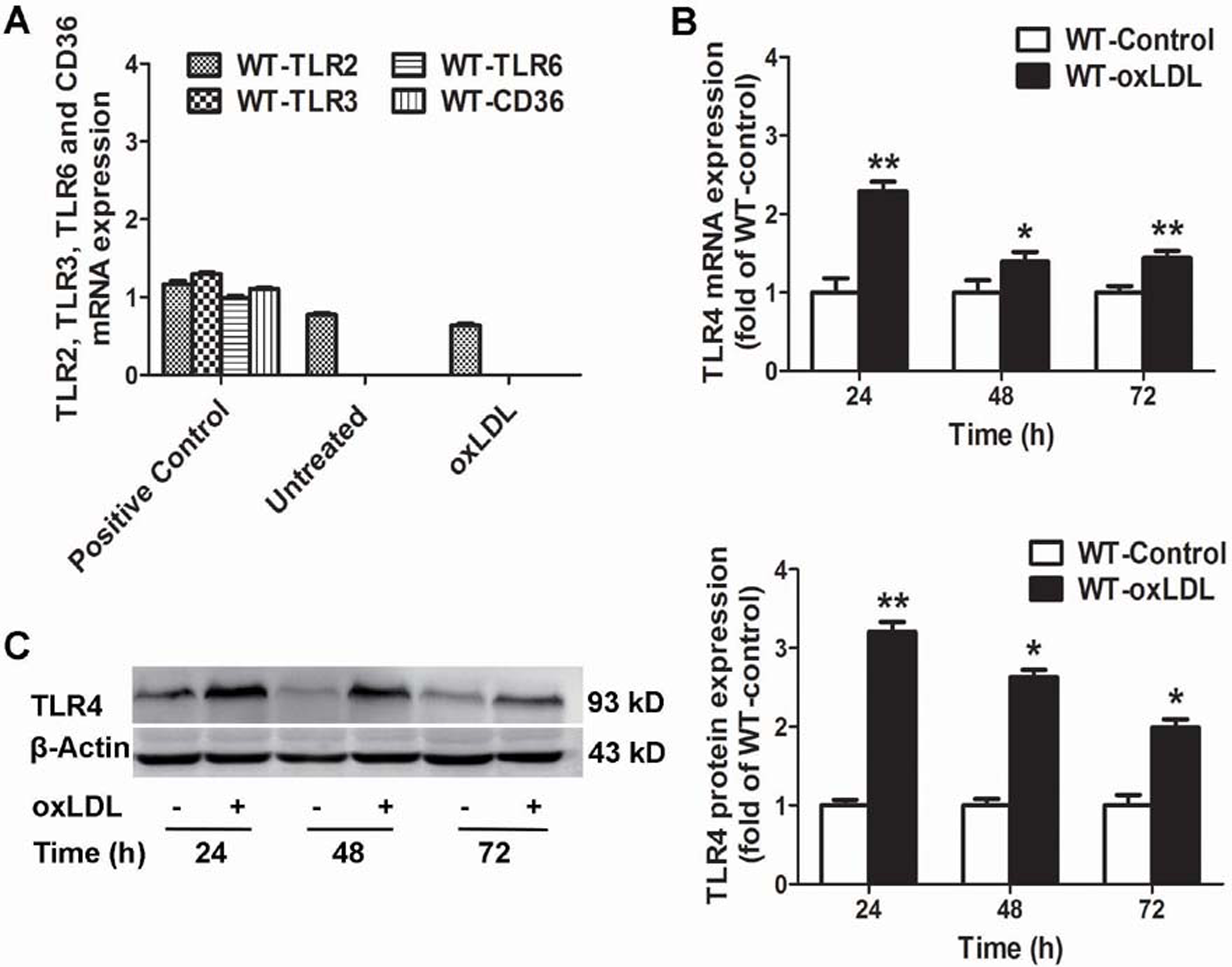

Supplement: Supplementary Figure 2 [file cddis2015212x2.tif]

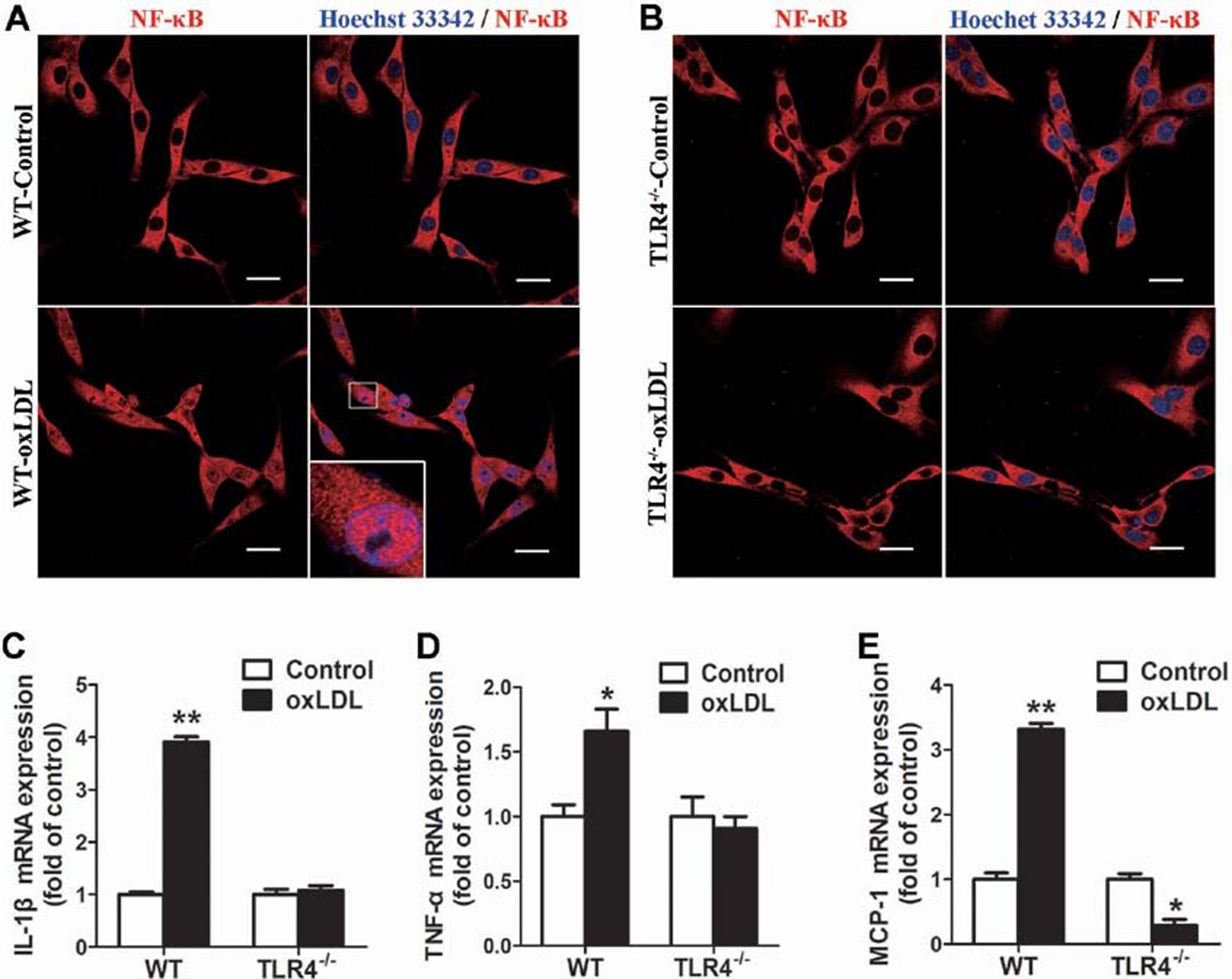

Supplement: Supplementary Figure 3 [file cddis2015212x3.tif]

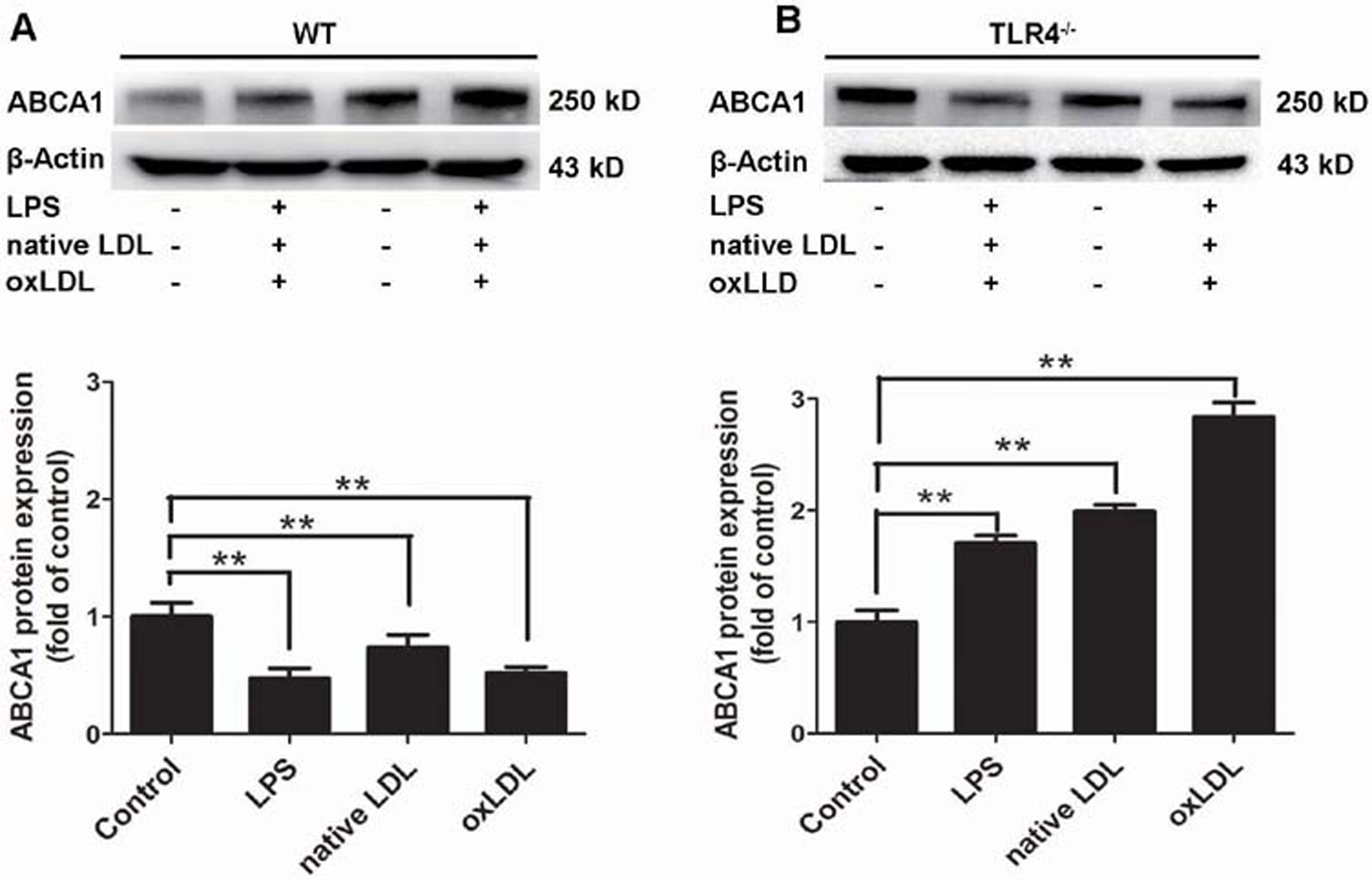

Supplement: Supplementary Figure 4 [file cddis2015212x4.tif]

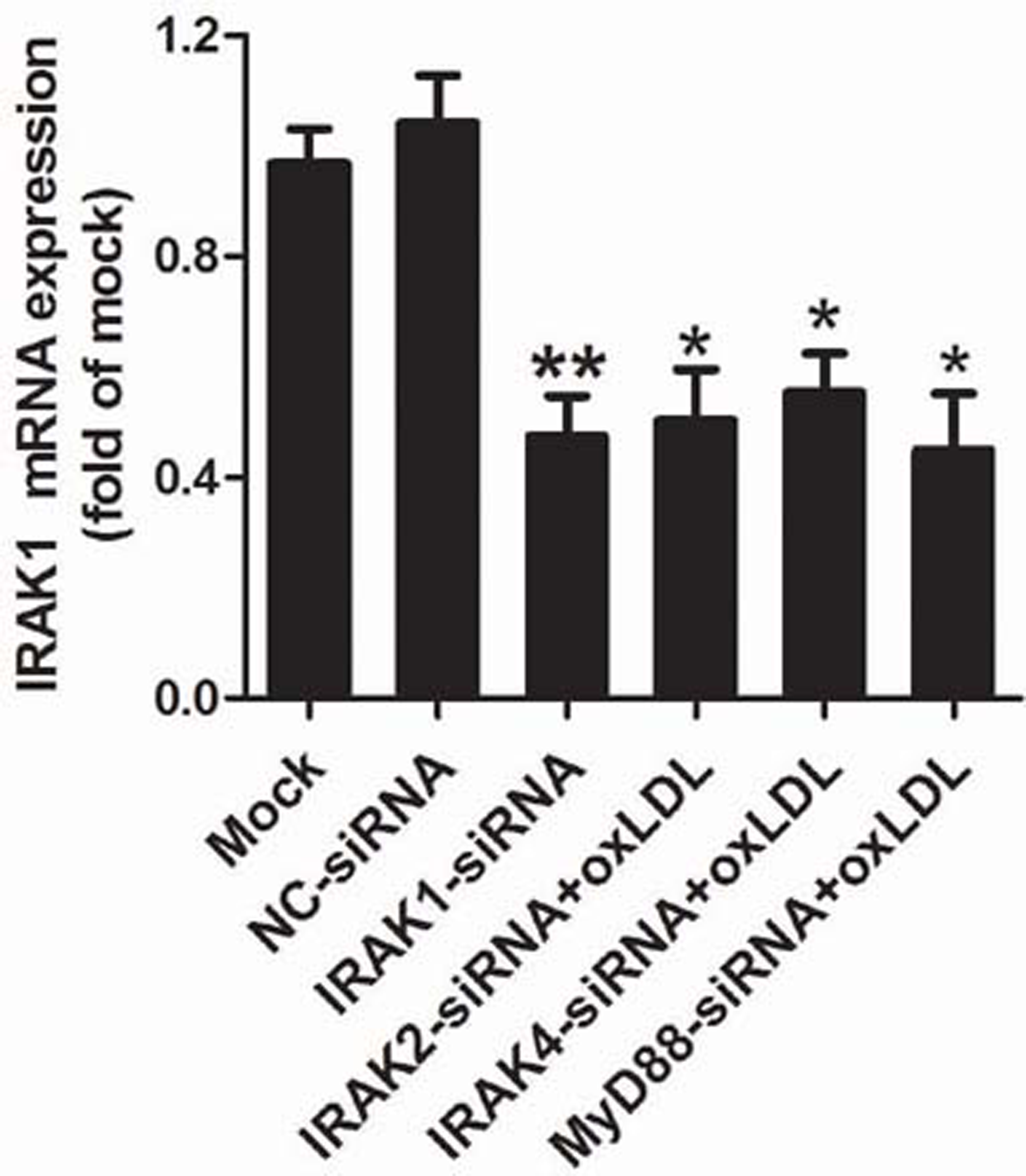

Supplement: Supplementary Figure 5 [file cddis2015212x5.tif]

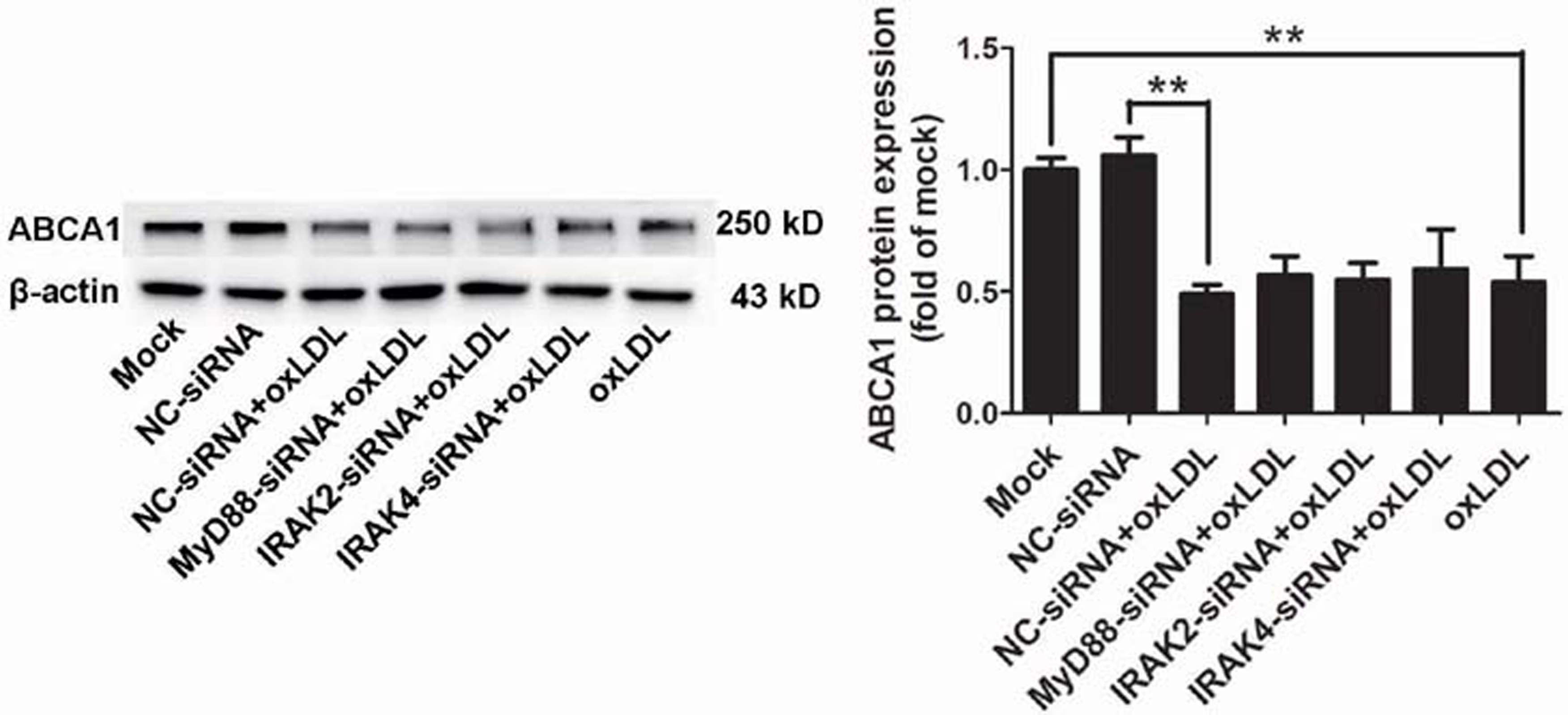

Supplement: Supplementary Figure 6 [file cddis2015212x6.tif]
