# Supplementary material for: IRAK1 mediates TLR4-induced ABCA1 downregulation and lipid accumulation in VSMCs
Source: Cell Death Dis. 2015 Oct 29;6(10):e1949–. doi: 10.1038/cddis.2015.212 (PMC5399175; doi:10.1038/cddis.2015.212)
Supplement: Supplementary Information [file cddis2015212x7.doc]

**Supplementary material for IRAK1 is critical for TLR4-induced ABCA1 downregulation and lipid accumulation in VSMCs**

**Lu Guo^1†^, Chun-Hai Chen^2†^, Li-Li Zhang^1†^, Xiao-Jie Cao^1^, Qin-Long Ma^2^, Ping Deng^2^, Gang Zhu^2^, Chang-Yue Gao^1^, Bing-Hu Li^1^, Yan Pi^1^, Yun Liu^1^, Zi-Cheng Hu^1^, Lei Zhang^2^, Zheng-Ping Yu^2^, Zhou Zhou^2^*, Jing-Cheng Li^1^***

^1^ Department of Neurology, Institute of Surgery Research, Daping Hospital, Third Military Medical University, Chongqing, People’s Republic of China. ^2^ Department of Occupational Health, Faculty of Preventive Medicine, Third Military Medical University, Chongqing, People’s Republic of China.

**^†^** These authors contributed equally to this work.

***** Co-correspondence: Jingcheng Li, MD, PhD, Department of Neurology, Institute of Surgery Research, Daping Hospital, Third Military Medical University, NO. 10 Changjiang Branch Road, Yuzhong District, Chongqing 400042, People’s Republic of China. Tel/Fax: +86 23 68757841; E-mail: lijingcheng11@aliyun.com. Zhou Zhou, PhD, Department of Occupational Health, Faculty of Preventive Medicine, Third Military Medical University, NO. 30 Gaotanyan Street, Shapingba District, Chongqing 400038, People’s Republic of China. Tel/Fax: +86 23 68752290; E-mail address: lunazhou00@163.com.

**Supplementary Table** **1.** Primers for real-time PCR

| Gene | Forward Primer, 5ˊ→ 3ˊ | Reverse Primer, 5ˊ→ 3ˊ |
| --- | --- | --- |
| *TLR4* | GCAGTTTCAATCGCATAGAGACAT | TGCTTCTGTTCCTTGACGCACT |
| *TNF-α* | TGACAAGCCTGTAGCCCACG | TGACAAGCCTGTAGCCCACG |
| *IL-1β* | ATGAAAGACGGCACACCCAC | TCTGCTTGTGAGGTGCTGATGTA |
| *ABCA1* | TCAGTTTGACGCCATGACAGAG | ACCAACCTTGCCAACTTCCTT |
| *MCP-1* | AGCACCAGCCAACTCTCACT | CGTTAACTGCATCTGGCTGA |
| *TLR2* | CGTTGTTCCCTGTGTTGCTG | GGATAGGAGTTCGCAGGAGC |
| *TLR3* | TACATCACGCAGTTCAGC | AGGCATTTATCCGTTCTT |
| *TLR6* | AACTCACCAGAGGTCCAA | TCTTCCCTGTCGATTCTC |
| *CD36* | GGCAGGAGTGCTGGATTA | GAGGCGGGCATAGTATCA |
| *β-actin* | CATCCGTAAAGACCTCTATGCCAAC | ATGGAGCCACCGATCCACA |

**Supplementary Figure 1** Identification of primary VSMCs. The VSMC phenotype was characterized by an immunofluorescence analysis of α-SMA (red fluorescent) and SM-22α (green fluorescent). Cells were co-stained with the myofibroblast marker vimentin (gray fluorescent). DAPI (blue fluorescent) was used to visualize nuclei. Scale bar = 20 μm.

**Supplementary Figure 2** TLRs and CD36 expression in WT VSMCs after oxLDL challenge. A, Cultured WT VSMCs were treated with or without 50 μg/mL oxLDL for 24 h. mRNA levels of TLR2, TLR3, TLR6 and CD36 were assessed by real-time PCR. Raw 264.7 macrophages were used as positive controls (n = 6 experiments in duplicate). B and C, Cultured WT VSMCs were treated with or without 50 μg/mL oxLDL for 24, 48 and 72 h. TLR4 mRNA and protein expression were determined by real-time PCR (n = 6 experiments in duplicate) and western blot (n = 3 experiments in duplicate). Data are expressed as the mean ± SEM. **P* < 0.05, ***P* < 0.01 compared with the untreated control group.

**Supplementary Figure 3** NF-κB nuclear translocation and pro-inflammatory cytokines expression in WT and TLR4**^－／－^** VSMCs in response to oxLDL. Cultured WT and TLR4**^－／－^** VSMCs were treated with or without 50 μg/ml oxLDL for 24 h. A and B, The subcellular location of the NF-κB p65 subunit was detected using an immunofluorescence assay. NF-κB p65 nuclear translocation was increased in oxLDL-treated WT VSMCs (A), but not in TLR4 knockout VSMCs (B). Hoechst 33342 (blue florescence) was used as a nuclear counterstain and indicated the nuclear localization of NF-κB p65 (red florescence). The white arrows show representative NF-κB nuclear translocated positive cells. Scale bar = 20 μm. C, D and E, Real-time PCR was performed to detect the expression of mRNAs that encode IL-1β, TNF-α and MCP-1 (n = 3 experiments in duplicate). All values are expressed as the mean ± SEM. **P* < 0.05, ***P* < 0.01 compared with the untreated control group.

**Supplementary Figure 4** The protein levels for ABCA1 in LPS, native LDL and oxLDL-treated WT and TLR4**^－／－^** VSMCs. Cultured WT and TLR4**^－／－^** VSMCs were treated with 100 ng/ml LPS, 50 μg/ml native LDL or 50 μg/ml oxLDL for 48 h. A western blot was performed to detect ABCA1 protein expression (n = 3 experiments in duplicate). Data are represented as fold changes relative to the controls. All values are expressed as the mean ± SEM. ***P* < 0.01 compared with the untreated control group.

**Supplementary Figure 5** Knockdown of IRAK1, IRAK2, IRAK4 or MyD88 mRNA levels by siRNA. WT VSMCs were transfected with negative control siRNA (NC-siRNA), IRAK1 siRNA, IRAK2 siRNA, IRAK4 siRNA or MyD88 siRNA. Experiments were performed 24 h after siRNA transfection. Efficiency in reducing IRAK1, IRAK2, IRAK4 or MyD88 mRNA levels was measured by real-time PCR (n = 4 experiments in duplicate). All values are expressed as the mean ± SEM. **P* < 0.05, ***P* < 0.01 compared to the same treatment in the mock setting.

**Supplementary Figure 6** Effects of MyD88, IRAK2 and IRAK4 on ABCA1 expression. Cultured WT VSMCs were transfected with negative control siRNA (NC-siRNA), MyD88 siRNA, IRAK2 siRNA or IRAK4 siRNA. Experiments were performed 24 h after siRNA transfection. Western blot was performed to detect ABCA1 protein expression (n = 3 experiments in duplicate). Data are represented as fold changes relative to the mock. Values are expressed as the mean ± SEM with ***P* < 0.01 compared to the same treatment in the mock setting.
